# Surfactant‐Mediated and Morphology‐Controlled Nanostructured LiFePO_4_/Carbon Composite as a Promising Cathode Material for Li‐Ion Batteries

**DOI:** 10.1002/open.201900175

**Published:** 2019-09-27

**Authors:** Sourav Khan, Rayappan Pavul Raj, Laurel George, G. S. Kamali Kannangara, Adriyan Milev, Upadhyayula V. Varadaraju, Parasuraman Selvam

**Affiliations:** ^1^ National Centre for Catalysis Research and Department of Chemistry Indian Institute of Technology-Madras Chennai- 600036 India; ^2^ School of Science and Health Western Sydney University Penrith NSW 2751 Australia; ^3^ Materials Science Research Centre and Department of Chemistry Indian Institute of Technology-Madras Chennai 600 036 India; ^4^ School of Chemical Engineering and Analytical Science The University of Manchester Manchester M13 9PL United Kingdom; ^5^ Department of Chemical and Process Engineering University of Surrey Guildford, Surrey GU2 7XH United Kingdom

**Keywords:** electrochemistry, carbon composites, cathode materials, carbon coating, Li-ion Batteries

## Abstract

The synthesis of morphology‐controlled carbon‐coated nanostructured LiFePO_4_ (LFP/Carbon) cathode materials by surfactant‐assisted hydrothermal method using block copolymers is reported. The resulting nanocrystalline high surface area materials were coated with carbon and designated as LFP/C123 and LFP/C311. All the materials were systematically characterized by various analytical, spectroscopic and imaging techniques. The reverse structure of the surfactant Pluronic® 31R1 (PPO‐PEO‐PPO) in comparison to Pluronic® P123 (PEO‐PPO‐PEO) played a vital role in controlling the particle size and morphology which in turn ameliorate the electrochemical performance in terms of reversible specific capacity (163 mAh g^−1^ and 140 mAh g^−1^ at 0.1 C for LFP/C311 and LFP/C123, respectively). In addition, LFP/C311 demonstrated excellent electrochemical performance including lower charge transfer resistance (146.3 Ω) and excellent cycling stability (95 % capacity retention at 1 C after 100 cycles) and high rate capability (163.2 mAh g^−1^ at 0.1 C; 147.1 mAh g^−1^ at 1 C). The better performance of the former is attributed to LFP nanoparticles (<50 nm) with a specific spindle‐shaped morphology. Further, we have also evaluated the electrode performance with the use of both PVDF and CMC binders employed for the electrode fabrication.

## Graphical abstract

**The Li‐Ion King**! Morphology‐controlled carbon‐coated nanostructured LiFePO4/Carbon composites prepared by surfactant‐assisted hydrothermal method show promise in terms of electrochemical performance with a reversible specific capacity of 163 mAh g^−1^ and 140 mAh g^−1^ (at 0.1 C rate) for LFP/C311 and LFP/C123 composite, respectively.

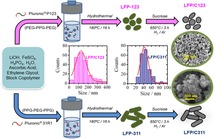

## 1 Introduction

LiFePO_4_ is a well‐established cathode materials for Li‐ion battery applications for its satisfactory electrochemical performances, low cost and good structural stability.^1^ It is not possible to exploit the full potential of the material due to its limited electrical conductivity and relatively slow diffusion of Li‐ions (∼10–18 cm^2^ s^−1^).^2^ LFP exhibits moderate theoretical specific capacity (∼170 mAh g^−1^) and redox potential (3.45 V versus Li^+^/Li). The primary focus of the recent research of nano‐sized LFP based cathode materials is to find out how to expeditiously improve the electrical conductivity of the olivine structured cathode material by advanced carbon coating strategies and hence facilitating faster Li‐ion transfer.^3^

Carbon coating is the usually applied straightforward method for ameliorating the conductivity of the cathode materials in general.^4^ The carbon coating process generally requires pyrolysis of sucrose on as‐synthesized LFP particles at high temperature under reducing atmosphere strictly.^5^ But this enhanced performance is derived at the cost of the volumetric energy density. Volumetric energy density is a very crucial factor and cannot be trivialized for practical Li‐ion batteries applications especially in case of electric vehicles.^6^ The surfactant assisted hydrothermal/solvothermal method used has a substantial influence on the particle morphology, microstructure, crystallite size, and textural properties of the prepared product.^7^ These properties by and large influence the electrochemical properties of the cathode material produced. Hydrothermal method can successfully produce nanoparticles with a more uniform nanostructure with controlled morphology which is an added advantage.^8^

Pluronic® P123 (M_n_∼5800) is a symmetric (ABA)‐type triblock copolymer of poly(ethylene glycol)‐block‐poly(propylene glycol)‐block‐poly(ethylene glycol) (PEO)_18_‐(PPO)_72_‐(PEO)_18_ with primary hydroxyl groups.^9^ Pluronic® 31R1 (M_n_∼3300) is a reverse (BAB)‐type triblock copolymer of poly(propylene glycol)‐block‐poly(ethylene glycol)‐block‐poly(propylene glycol) (PPO)_26_‐(PEO)_5_‐(PPO)_26_ with terminal secondary hydroxyl groups.^10^ Terminal secondary hydroxyl groups have comparatively lower reactivity than the primary hydroxyl group in case of block copolymer based surfactants. Pluronic® 31R1 has very few hydrophilic PEO blocks compared to Pluronic® P123 triblock copolymer. Pluronic® 31R1 has reduced gelling tendencies relative to that of Pluronic® P123 surfactant. It is to be noted that surface activity of block copolymer based non‐ionic surfactants generally depends on the ratio of hydrophilic (EO)/hydrophobic (PO) units present.^11^ The Pluronic® 31R1 contains only 10 wt.% PEO whereas Pluronic® P123 contains 30 wt.% PEO. In aqueous solution Pluronic® P123 forms micelles where the hydrophobic core generally constitutes PPO block and a hydrophilic corona is comprised of PEO block.^12^ The terminal hydrophilic rich PEO group is expected to form more stable micells in Pluronic P123.

Here, we report synthesis of nano‐LFP/Carbon composite using tri‐block copolymer‐based surfactants such as Pluronic® 31R1 by hydrothermal method for the first time. We also synthesized the nano‐LFP/Carbon composite using P123 surfactant and compared the electrochemical performance with the former.

## 2 Results and Discussion

**Figure 1:**
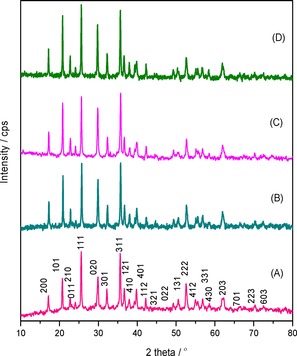
Powder X‐ray diffraction patterns of: (A) LFP‐123 (B) LFP‐311 (C) LFP/C123 and (D) LFP/C311.

**Figure 2:**
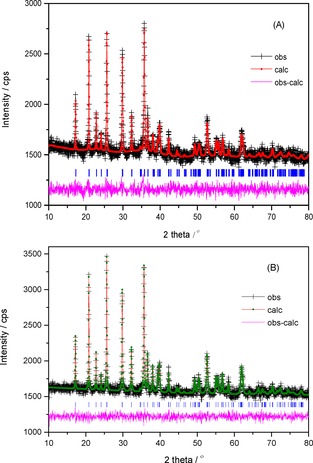
The Rietveld refinement of: (A) LFP/C123 and (B) LFP/C311.

Powder XRD patterns of both the as‐synthesised (LFP‐123 and LFP‐311) and the calcined LFP/Carbon (LFP/C123 and LFP/C311) nanocomposites are shown in Figure 1. The observed XRD diffraction patterns of all the samples can be indexed to orthorhombic olivine‐structured LiFePO_4_ (JCPDS Card No: 83‐2092) with *Pnma* space group. It is to be noted here that no impurity secondary phases such as Fe_2_P_2_O_7_ or Fe_2_P are observed.^13^ Rietveld refinement analysis of the XRD patterns of LFP/Carbon composites (Figure 2) were performed using GSAS (General Structure Analysis System). The background was fitted with a shifted Chebyschev polynomial function and various crucial parameters such as zero point, scale factor, profile parameters, atomic positions, and coefficients for the peak shape function were refined until convergence is reached. (R_*p*_=1.7 % and R_*wp*_=2.2 % for LFP/C311 sample).^14^ The computed lattice constants for both the samples are tabulated in Table 1 and are in good agreement with values reported in literature.^15^ Further the LFP phase is free from antisite defects as the unit cell volumes is around ∼290 Å^3^ which is considered to be a good measure of lack of antisite defects.^16^

**Table 1:** Structural properties of LFP/Carbon composites.

Material	*a*	*b*	*c*	*V*	*R_p_*	*R_wp_*	χ^2^
	(Å)	(Å)	(Å)	(Å)^3^	(%)	(%)	
LFP/C123	10.305(9)	5.995(5)	4.701(4)	290.442(2)	1.69	2.16	0.74
LFP/C311	10.332(1)	5.993(5)	4.696(4)	290.820(3)	1.96	2.58	1.06

**Figure 3:**
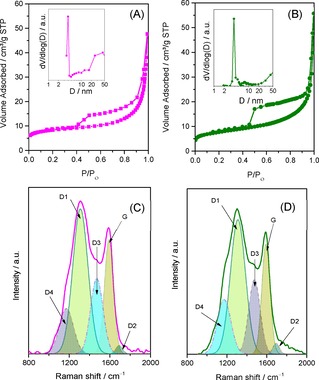
N_2_ sorption isotherms of: (A) LFP/C123 and (B) LFP/C311. The inset shows the corresponding PSD. Raman spectral signature of: (C) LFP/C123 and (D) LFP/C311.

Figure 3(A–B) shows the typical N_2_ sorption isotherms and its corresponding BJH pore size distribution curve of both the LFP/Carbon composites. LFP/C311 sample shows a typical type IV isotherm with H3‐type hysteresis loop and the measured specific surface area of the composite is 29 m^2^ g^−1^. The estimated pore size and pore volume were 3.8 nm and 0.07 cm^3^ g^−1^, respectively. The composites were calcined in a mildly reducing atmosphere (5 % H_2_/Ar), and the carbon left in the sample owing to pyrolysis of sucrose also contributed to the surface area of the composite significantly.^17^ Textural properties of the LFP/Carbon composites are tabulated in Table 2. From CHN analysis the carbon content values in LFP/C311 and LFP/C123 composite were found to be 4.7 and 4.5 wt.%, respectively.

**Table 2:** Textural and spectral properties of LFP/Carbon composites.

Material	BET Data			Raman Data		
	*S_BET_*	*D_BJH_*	*V_P_*	*G*	*D1*	*D1/G*
	(m^2^ g^−1^)	(nm)	(cm^3^ g^−1^)	(cm^−1^)	(cm^−1^)	
LFP/C123	25	3.86, >25	0.07	1592	1304	2.34
LFP/C311	29	3.81, >20	0.07	1591	1302	2.27

Raman spectroscopy is a non‐destructive diagnostic tool to assess the quality of the carbon coating on olivine‐based cathode materials. Figure 3 (C–D) depicts the Raman spectra for both the LFP/Carbon composites. As anticipated, the Raman spectra is typically comprised of two intense signals corresponding to *D*‐band (∼1300 cm^−1^) and *G*‐band (∼1590 cm^−1^) of coated carbon in both cases. These can be further de‐convoluted into five peaks.^18^ The *G*‐band is specifically related to the *E_2g_* zone center mode of crystalline graphite.^19^ The broad *D1*‐band at 1300 cm^−1^ is generally^20^ assigned to the vibrational mode with *A_1g_* symmetry of disordered graphitic lattice. The *D2* band which appeared as a shoulder on the *G*‐band represents a highly defective graphitic lattice mode having *E_2g_* symmetry. The *D3* band for both the composites at ∼1480 cm^−1^ is assigned to amorphous carbon. The *D4* band at ∼1175 cm^−1^ is associated with diamond‐like carbon with short‐range vibrations of *sp* ^*3*^‐carbon. The ratio of the integrated area of the *D1*‐band and *G*‐band (A_*D1*_/A_*G*_) is used to evaluate the degree of disorder. For the LFP/C311 composite the value is 2.27. This unequivocally proves that the uniform carbon coating of LFP/C311 composite contains large amount of graphene domains.

**Figure 4:**
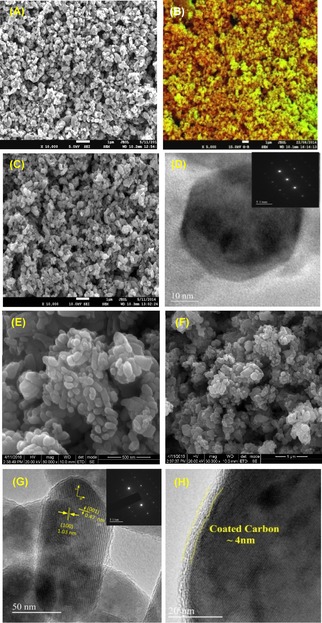
(A–B) SEM image of LFP/C123, (C) False coloured SEM image of LFP/C123; (D) TEM image and SAED patterns (inset) of LFP/C123; (E–F) SEM image of LFP/C311; (G) TEM images and SAED patterns (inset) of LFP/C311; (H) HR‐TEM image showing the average carbon coating thickness of 4 nm around LFP nanoparticles.

In order to investigate morphology as well as the microstructure of LFP/Carbon composite, we have carried out electron microscopy studies. Figure 4 depicts the SEM and TEM images of LFP/C123 and LFP/C311 composites, respectively. The particles are mostly clustered and sintered (Figure 4A) with no distinct boundaries for the LFP/C123 sample. The false‐coloured SEM image (*see* Figure 4C) shows some particles have a red glow to them, suggesting these are fully carbon coated. On the other hand, the SEM images of LFP/C311 sample (Figure 4E) shows particles with spindle shaped morphology with particle width less than 50 nm. The primary particle size of LFP/C311 is much smaller than LFP/C123 (*see* Figure 4A & 4F). The width of the LFP/C311 is centered at around 50 nm while there is a broad particle size distribution is noticed in the case of LFP/C123. In order to support the same, we have presented the TEM image of LFP/C123 (*see* Figure 4D). The TEM image (Figure 4G) clearly reveals that a fairly uniform carbon layer formed over the LFP nanoparticles with a thickness of 3–4 nm in case of LFP/C311 composite. As depicted in Figure 4G, the interplanar spacing of 1.03 nm and 0.43 nm corresponds to the (100) and (001) lattice planes, respectively. Hence, the exposed crystallographic facet of the nano‐spindle is the (010) plane. The SAED patterns for both the composites (Inset Figure 4D and G) show a bright spot pattern typical of crystalline LiFePO_4_.

**Scheme 1:**
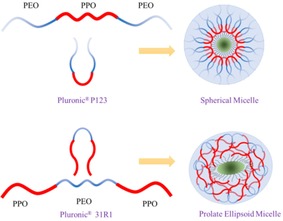
Representation of micelle formation and microstructure controlled by tri‐block co‐polymer based surfactants.

Based on the cloud point, hydrophilic‐libophilic balance (HLB)^16^ and solubility in water, the micelle formation tendency and solvation of Pluronic® P123 is greater than Pluronic® 31R1. At the LFP interface both the surfactants form thin layers; consequently the interfacial area covered by Pluronic® 31R1 is greater than Pluronic® P123, because 72 and 52 hydrophobic (PPO) units in case of Pluronic® P123 and Pluronic® 31R1 respectively remain at the LFP interface, by way of folding and orientations guided mainly by steric restriction. The morphology of the LFP aggregates in case of Pluronic® P123 surfactant is more or less spherical whereas Pluronic® 31R1in turn produces somewhat spindle shape, as revealed by the respective HRTEM images. The Pluronic® P123 sample assemblies grew in size by way of coalescence during temperature raising. In general, at higher temperatures, the micelle core radius increases steadily and when the core radius surpasses the stretched length of core blocks, the micelles incline to change the shape from spherical to prolate ellipsoid or rod like‐cylindrical structures.^21^ Furthermore, when the temperature of a block copolymer solution is elevated, the PPO block increasingly loses its hydration sphere, but the PEO blocks retain their strong interaction with water to certain extend.^9^, ^22^ The anisotropic growth of the hydrophobic core with raising temperature due to the increasing dehydration of PEO blocks in the corona induces instability in the spherical micellar dispersion, contributing largely to the formation of rod‐like cylindrical structures in case of Pluronic 31R1 surfactant. But in case of Pluronic P123 surfactant larger amount of hydrophilic PEO units are present in the corona and hence the stable spherical micelles remain intact even at higher hydrothermal temperature (*see* Scheme 1). Normally, the reverse tri‐block co‐polymer micelles prefer an oval‐shaped ellipsoidal structure in contrast to sphere and the interaction between micelles is predominantly repulsive.^23^ Moreover, the uniformity in size^24^ and surface homogeneities largely depends on the surface tension directed self‐assembly in the microstructure size domain in case of Pluronic® 31R1 surfactant. There is a prominent difference between the aggregation behavior of both the surfactants, where Pluronic® P123 aggregates became nearly monodisperse at elevated temperature in contrast to Pluronic® 31R1. As the number‐average molecular weight of Pluronic® 31R1 is less, hence it forms relatively thin carbonaceous layer on the surfaces of the spindle shaped crystalline LiFePO_4_ nanoparticles. Hence, there is a difference in morphology of the LFP nanoparticles.

**Figure 5:**
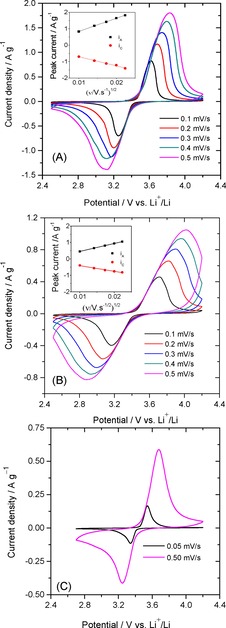
Cyclic voltammograms of: (A) LFP/C123 (PVDF), (B) LFP/C311(PVDF)and LFP/C311 (CMC) at different scan rates. Inset: Relationship between the peak currents and ν^1/2^ at various scan rates.

Cyclic voltammogram (CV) experiments were performed at various scan rates (0.1–0.5 mV s^−1^), in order to examine the redox processes occurring in LFP/C123 and LFP/C311 composites in the potential range of 2.5–4.2 V (vs. Li^+^/Li). As shown in Figure 5(B), two sharp redox reaction peaks are observed at 3.65 V and 3.2 V vs Li^+^/Li at a scan rate of 0.1 mV s^−1^ for the LFP/C311 nanocomposite, which correspond to Li‐ion de‐intercalation/intercalation process. But in case of LFP/C123 electrode the redox peaks are broad which depicts poor reactivity in comparison to LFP/C311 composite electrode. This can be mainly attributed to relatively poor electronic conductivity resulting from improper carbon coating (see false colored SEM image Figure 4 (C)) over LFP nanoparticles. With the intention of estimating the Li‐ion diffusion coefficient, we plotted the peak current at different sweep rates as shown in Figure 5 (inset).

Furthermore the peak current has a linear relationship with the square root of scan rate according to Randles‐Sevcik equation:

(1) $$i{}_{p}=2.69\times 10{}^{5}n{}^{3/2}\mathrm{AC}{}_{0}D{}_{0}{}^{1/2}v{}^{1/2}$$

where *i_p_* is the peak current (A), n is the number of electrons involved in the redox process, *C_0_* is the bulk concentration of lithium in the electroactive material (2.2×10^−2^ mol.cm^−3^), *ν* is the scan rate and A is the electrochemical active surface area of the electrode. The estimated anodic and cathodic diffusion coefficients for LFP/C123 composite are 1.81×10^−12^ and 9.14×10^−13^ cm^2^ s^−1^, respectively, and the corresponding values for LFP/C311 composite are 4.97×10^−12^ and 2.34×10^−12^ cm^2^ s^−1^, respectively.

**Figure 6:**
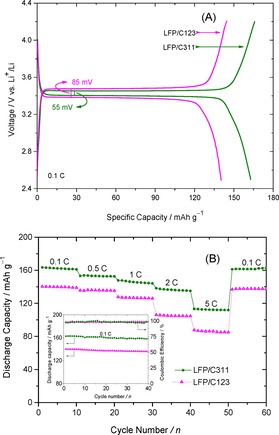
Galvanostatic charge‐discharge profiles for: (A) LFP/C123 and LFP/C311 at 0.1 C; (B) The rate performance of LFP/Carbon electrodes at different current rates. Inset: Cyclic performance of LFP/C123 and LFP/C311 composites at a current rate of 0.1 C.

The initial galvanostatic charge/discharge cycling profiles of both the LFP/Carbon composites at a current rate of 0.1 C and voltage range of 4.2–2.5 V is shown in Figure 6(A).The LFP/C311 composite electrode delivered a first discharge capacity of 163 mAh g^−1^ which is about 97.2 % of the theoretical capacity. Furthermore, LFP/C311 composite electrode exhibits superior rate capability performance vis‐à‐vis LFP/C123, with very little capacity fading and the discharge capacities value are are 154, 147, 136 and 112 mAh g^−1^ at 0.5 C, 1 C, 2 C and 5 C respectively, as shown in Figure 6(B). But, in case of LFP/C123 composite the corresponding discharge capacity values are significantly low when cycled at various C‐rates.When the LFP/C311 electrode cycled at 5 C rate was dischared at lower current rate (1 C) nearly 99 % of the initial discharge capacity was recovered indicating the composite electrode can sustain high rate charge/discharge. Again, the specific capacity of LFP/C311 composite at moderate C‐rate is comparable to previously reported LiFePO_4_ nanoparticles prepared using various surfactant‐assisted hydro or solvothermal methods. (*see* Table 3).

**Table 3:** Comparison of Electrochemical Performance of LFP prepared using various surfactant‐assisted hydro/solvothermal methods.

Surfactant	Material	Particle Size	Specific Capacity	Ref.
Cationic Template				
CTAB	LFP	50 nm	140 mAh g^−1^ @ C/10	[25]
CTAB	LFP/C	2000–5000 nm	120 mAh g^−1^ @ C/10	[26]

Anionic Surfactant				
SDS	LFP	100 nm	158 mAh g^−1^ @ C/10	[27]
SDBS	LFP	200–500 nm	146 mAh g^−1^ @ 1 C	[28]
SDBS+[BuMIm][BF_4_]	LFP	200 nm	160 mAh g^−1^ @ C/20	[29]
Avanel S‐150	LFP	<100 nm	132 mAh g^−1^ @ C/10	[30]

Non‐ionic Surfactant				
Tween 40	LFP/C	73 nm	155 mAh g^−1^ @ C/10	[31]
Triton X‐100	LFP	80–200 nm	140 mAh g^−1^ @ C/10	[32]
Brij‐78	LFP	78 nm	158 mAh g^−1^ @ C/12	[33]
PVP	LFP	50 nm	110 mAh g^−1^ @ C/30	[34]

Note: CTAB – cetyl trimethyl ammonium bromide; SDS – sodium dodecyl sulfonate; Tween 40 – Polyoxyethylenesorbitan monopalmitate; Triton X‐100 – isooctylphenylether of polyoxyethylene; [BuMIm][BF_4_] – 1‐butyl‐3‐methylimidazolium tetrafluoroborate; Avanel S‐150 – sodium C_12‐15_ alkyl polyoxyethylene sulfonate; SDBS – sodium dodecyl benzene sulfonate; PVP – polyvinylpyrrolidone.

**Figure 7:**
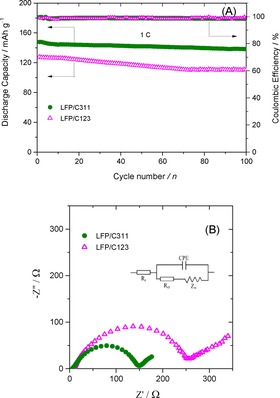
(A) The cyclic performance of LFP/Carbon composite electrodes; (B) Nyquist plots of LFP/C123 and LFP/C31.

Even after 40 cycles, a reversible capacity of 158 mAh g^−1^ was observed (Figure 6(B) inset) at 0.1 C rate suggesting the good cyclic stability and reversibility in case of LFP/C311 electrode. Furthermore, LFP/C311 nanocomposite demonstrates excellent cycling performance (Figure 7(A)) with capacity retention of over 95 % after 100 cycles at 1 C rate, while LFP/C123 composite electrodes show relatively poor cycling performance with capacity retention of 87.4 % after 100 cycles at 1 C rate. The decent retention of capacity, superior cyclic performance and high coulombic efficiency could be attributed to high electronic conductivity resulting from the thin and uniform carbon layer coating over LFP nanoparticles for the LFP/C311 composite.

**Figure 8:**
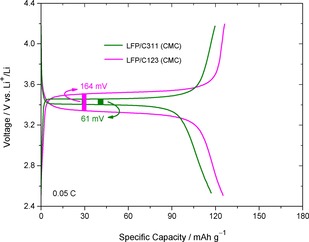
Galvanostatic charge‐discharge profiles for LFP/C123 (CMC) and LFP/C311 (CMC) composite at 0.05 C.

In order to explore the possibility of using environmentally benign process, LFP/Carbon composites were fabricated using CMC as binder in aqueous medium and no additional conductive carbon such as acetylene black, Super P etc. has been added during the preparation of the slurry as we are trying to utilize just the carbon present from synthesis. Hence, there is always a risk of losing some capacity because of the presence of ppm level of water which can be detrimental to the overall cell performance although huge care has been taken during the drying procedure.^35^ Also the immersion duration has been reduced to minimum in order to limit the aging of LiFePO_4_ during aqueous electrode processing.^36^ The charge discharge profile of LFP/C123 and LFP/C311 electrodes fabricated in aqueous medium using CMC binder is given in Figure 8. The initial capacity of the LFP/C123 electrode fabricated using (CMC) as binder is considerably lower than that of the PVDF binder. The flat voltage profile and reasonable capacity values indicate that the cheaper, greener, and easier to handle CMC could be successfully utilised as an optional binder for Li‐ion batteries.

In order to study the charge transfer resistance we carried out the electrochemical impedance spectroscopy studies as shown in Figure 7(B). From the Nyquist plots it can be easily visualized that the diameter of the semicircle of LFP/C311 electrode was found to be smaller than that of the LFP/C123 electrode indicating that the LFP/C311 electrode possesses a substantially lower charge transfer resistance. Both the materials exhibit a sloping straight line in the low‐frequency region, which is generally attributed to the Li‐ion diffusion into the bulk of the electrode material.^37^ The plots are fitted using equivalent circuit given in Figure 7(B) and various parameters were reduced.

In case of LFP/C311 electrode the solution resistance is 6 Ω, charge‐transfer resistance is 146 Ω, double layer capacitance of 13.8 μF and the Warburg impedance is within the range of 7 Hz to 0.1 Hz. The Li‐ion diffusion coefficient (D_Li+_) in LFP/Carbon electrode could be computed by the equation given below:^38^

(2) $$D{}_{\mathrm{Li}+}=\left(R{}^{2}T{}^{2}\right)/\left(2A{}^{2}n{}^{4}F{}^{4}C{}^{2}\sigma {}^{2}\right)$$

where *R* is the gas constant, *T* is the absolute operating temperature, *A* is the surface area of the electrode, *n* is the number of electrons per molecule during oxidation process, *F* is Faraday's constant, and *C* is the molar concentration of Li‐ions (7.69×10^−3^ mol cm^−3^), and *σ* is the Warburg factor obtained from the slope of the plot of Z_real_ versus the reciprocal square root of angular frequencies (ω^−1/2^). In case of the LFP/C311 composite, the calculated Li‐ion diffusion coefficient (D_Li+_) value is approximately one order of magnitude higher than that of the LFP/C123 composite (*see* Table 4); these results clearly indicate that the uniform thin carbon coated LFP/C311 electrode succeeds in ameliorating the mobility of lithium ion transport pathways and hence, the sample demonstrates better cyclic performance.

**Table 4:** Electrochemical parameters of LFP/Carbon composites.

Material	Capacity (mAh g^−1^)^[a]^		*D*_Li_+ (cm^2^ s^−1^)			*R_CT_* (Ω)
	1^st^ cycle	50^th^ cycle	Cathodic^[b]^	Anodic^[b]^	EIS	
LFP/C123	140.1	134.9	9.1×10^−13^	1.8×10^−12^	7.2×10^−14^	250.4
LFP/C311	163.3	158.1	2.3×10^−12^	4.9×10^−12^	5.6×10^−13^	146.3

[a] Discharge capacity at 0.1 C rate. [b] Diffusion coefficient calculated from cyclic voltammogram.

## 3 Conclusions

In conclusion, Pluronic® 31R1 surfactant plays a vital role in regulating the morphology and generating controlled nano‐sized LFP particles during hydrothermal synthesis. On the other hand, in case of Pluronic® P123 based surfactant, specific control of LFP particle size and morphology is not possible simultaneously.The LFP/C311 sample shows enhanced electrochemical performance in terms of good reversible capacity, high rate capability, and cycling stability. The reasonably good performance of LFP electrodes fabricated in aqueous medium with CMC as a binder paves a way for cleaner greener and environmentally benign fabrication technology of LFP based Li‐ion batteries.

## Experimental Section

**Starting Materials**: All the chemicals used in this study were analytical grade reagents without any further purification. The surfactants (Pluronic® P123 and Pluronic® 31R1) were obtained from Sigma‐Aldrich. The raw materials LiOH ⋅ H_2_O (purity >99.95 wt.%), FeSO_4_ ⋅ 7H_2_O (ACS reagent, ≥99.0 %), H_3_PO_4_ (≥85 wt.% in H_2_O) were procured form Sigma‐Aldrich directly. Ethylene glycol (anhydrous, 99.8 %) and ascorbic acid (reagent grade) were acquired from Merck and used as received. Deionized water is used strictly in the experiment.

**Synthesis**: LFP/Carbon composite was synthesized by a two‐step procedure via surfactant assisted hydrothermal method from the stoichiometric mixture of LiOH, FeSO_4_ ⋅ 7H_2_O and H_3_PO_4_ (3 : 1 : 1 molar ratio). Block copolymers viz., Pluronic® 31R1 and Pluronic® P123 were employed as structure directing agent. At first, the tri‐block copolymer and ascorbic acid were added to the stoichiometric starting materials constantly dissolved in water. Ethylene glycol was then added to the aqueous solution so that a greyish green gel was obtained. The resulting greenish green gel was transferred into a 150 mL teflon‐lined stainless steel autoclave and heated at 180 °C for 16 h. The olive green solid product was completely washed and dried at 80 °C, and designated as LFP‐123, and LFP‐311, respectively for the samples prepared with Pluronic® P123 and Pluronic® 31R1 surfactants. The as‐synthesized LFP‐123 and LFP‐311 composites were mixed with 20 wt.% sucrose solution and calcined at 650 °C for 3 h under H_2_/Ar gas atmosphere. The final samples prepared were designated as LFP/C123 and LFP/C311 respectively.

**Characterization**: All the samples including LFP‐123 and LFP‐311 were systematically characterized via various analytical, spectroscopic and imaging techniques. A Bruker D8 Advance X‐ray diffractometer with 0.02°/step and a step time of 5 sec/step using Cu Kα (λ=1.54056 Å) radiation was used to determine the phases as well as Rietveld refinement of the X‐ray diffraction patterns was performed to obtain precise structural parameters using general structure analysis system (GSAS). Scanning electron micrographs were collected using a JOEL 7001F microscope. False colour images were made by taking image using the secondary detector and a second image using the backscatter detector in composition mode. Each image was assigned a colour (red or green), and the images were then combined. Because backscattered images of carbon are darker than the LFP, they appear as different colours in the false coloured image. Transmission Electron Microscope (TEM) analysis was performed by JEOL JEM 2010 electron microscope operated at 200 keV with a point‐to‐point resolution of 2.3 Å. Analysis of BET surface area was undertaken on a Micrometrics ASAP 2020. Samples were first degassed under vacuum at 200 °C for 48 h before measurements and then backfilled with He. Any trapped He was removed from sample pores just prior to analysis by further degassing under vacuum at 200 °C. The adsorption/desorption isotherms were carried out under N_2_ at 77 K. Analysis for carbon content in the calcined sample was carried out using PerkinElmer 2400 Series II CHNS/O elemental analyser. Raman spectra of the powders were recorded using a BrukerSenterra III Raman spectrometer equipped with a 532 nm excitation laser with power kept to <0.2 mW. During measurements, the laser beam was focused through an objective lens with 50×magnification, and an aperture of 50×1000 μm.

**Electrode Fabrication**: Electrochemical measurement was carried out by the assembly of two‐electrode Swagelok‐type cell inside an argon‐filled glove‐box (mBraun, <0.1 ppm H_2_O and O_2_<0.6 ppm). For the fabrication of cathode, the active material (LFP/C123 and LFP/C311), Super P carbon black and poly(vinylidenedifluoride) (PVDF) were used in the weight ratio 80 : 10 : 10 and grounded homogenously using N‐methyl‐2‐pyrrolidinone (NMP) solvent and the resultant slurry was coated on an aluminum foil (φ=12 mm) and subsequently dried in an air oven at 80 °C for 12 h. The foil was cut into circular discs with an area of 0.8 cm^2^ and the loading of the active material on the aluminum foil was ∼2 mg cm^−2^. 1 M LiPF_6_ dissolved in a mixture of ethylene carbonate (EC) and dimethyl carbonate (DMC) (1 : 1 vol %) was used as electrolyte and Whatman glass microfiber (GF/D) was used as the separator. Lithium foil with a thickness of <0.5 mm was used as both the reference and counter electrode. The cells were allowed to equilibrate for 24 h at room temperature. Subsequently, the cells were galvanostatically charged and discharged in the voltage window of 2.5–4.2 V vs Li^+^/Li at room temperature using Arbin battery testing system (Model BT2000, USA). Cyclic voltammograms (CV) were recorded using Autolab instrument in the potential range of 2.5–4.2 V vs. Li^+^/Li at various scan rates ranging from 0.1 to 1 mV s^−1^. The electrochemical impedance spectra (EIS) measurements were carried out using Bio‐logic VSP instrument.

In one case the LFP/Carbon were used for fabrication with no additional Super P carbon added to electrode slurries. Electrode slurries were formed by mixing LFP/Carbon sample and the binder CMC (low viscosity sodium carboxy methyl cellulose) at a ratio of 16 : 1 respectively. The solvent, (water and ethanol in a 50 : 50 ratio by volume) was added until the slurry reached an appropriate viscosity. Slurries were then mixed in a planetary ball mill for 40 minutes at 100 rpm with 10×5 mm and 1×20 mm zirconium oxide balls. Electrodes were formed by spreading the prepared slurry onto a lightly sanded and cleaned aluminium foil current‐collector with a K‐bar (Revco), resulting in a 60 μm thick wet film, and left to air dry until touch‐dry. Electrodes then underwent the following drying regime: 1) air‐dried until touch‐dry in a fume hood, 2) a drying oven at 100 °C for 1 hour, 3) a vacuum oven (0.5 bar) at 100 °C for 1 hour, and 4) a drying oven at 70 °C oven for 1 hour. Electrodes were then stored in a desiccator until ready for battery preparation. The cells were assembled in a glove‐box under an argon atmosphere with oxygen and water contents both maintained below 2 ppm. The cathode was prepared by cutting circular disks from the prepared electrode sheets. The anode consisted of a 19 mm diameter lithium metal disk, which also served as a reference electrode. A 1 M solution of LiPF_6_ in ethylene carbonate (EC) and dimethyl carbonate (DMC) mixture (1 : 1 w/w) was used as the electrolyte and a Celgard 2500 polypropylene (PP) membrane as the separator. The electrodes fabricated using the CMC binder were designated as LFP/C123 (CMC) and LFP/C311 (CMC) during electrochemical measurements.

**Electrochemical measurements**: 1 M LiPF_6_ dissolved in a mixture of ethylene carbonate (EC) and dimethyl carbonate (DMC) (1 : 1 vol %) was used as electrolyte and Whatman glass microfiber (GF/D) was used as the separator. Lithium foil of thickness less than 0.5 mm was used as both the reference and counter electrode. The cells were allowed to equilibrate for 24 h at room temperature (RT). The cells were galvanostatically charged and discharged in the voltage window of 2.7–4.0 V versus Li^+^/Li at room temperature using Arbin battery testing system (Model BT2000). Cyclic voltammograms (CV) were recorded using Autolab instrument in the potential range of 2.5–4.2 V vs. Li^+^/Li at various scan rates ranging from 0.1 to 0.5 mV s^−1^. The impedence studies were carried out using a BioLogic VSP instrument.

## Conflict of interest

Indian patent application is pending for inventors P. Selvam, S. Khan and R.P. Raj (Provisional Number: 201841047364).
